# Echocardiographic Assessment of Right Ventricular–Pulmonary Arterial Coupling in Heart Failure: Prognostic Insights from a Systematic Review

**DOI:** 10.3390/jcm15062334

**Published:** 2026-03-18

**Authors:** Andrea Sonaglioni, Michele Lombardo, Giulio Francesco Gramaglia, Gian Luigi Nicolosi, Alessandro Lucidi, Massimo Baravelli, Sergio Harari

**Affiliations:** 1 Division of Cardiology, IRCCS MultiMedica, 20123 Milan, Italy; michele.lombardo@multimedica.it (M.L.); massimo.baravelli@multimedica.it (M.B.); 2 Department of Emergency, Fondazione IRCSS Ca’ Granda, Ospedale Maggiore Policlinico, 20122 Milan, Italy; giulio.gramaglia@unimi.it; 3 Division of Cardiology, Policlinico San Giorgio, 33170 Pordenone, Italy; gianluigi.nicolosi@gmail.com; 4 Semi-Intensive Care Unit, Division of Pneumology, IRCCS MultiMedica, 20123 Milan, Italy; alessandro.lucidi@multimedica.it (A.L.); sergio.harari@unimi.it (S.H.); 5 Department of Clinical Sciences and Community Health, Università di Milano, 20122 Milan, Italy

**Keywords:** heart failure, right ventricular–pulmonary arterial coupling, TAPSE/sPAP, echocardiography, right ventricular function, prognosis

## Abstract

**Background**: Prognostic heterogeneity in heart failure (HF) is substantial and not fully captured by conventional left-sided echocardiographic parameters. Growing evidence highlights the importance of right ventricular–pulmonary arterial (RV–PA) interaction in HF pathophysiology and outcomes. The echocardiographic tricuspid annular plane systolic excursion-to-systolic pulmonary artery pressure (TAPSE/sPAP) ratio has been proposed as a simple noninvasive surrogate of RV–PA coupling, yet its prognostic value across the HF spectrum remains incompletely defined. **Methods**: This systematic review followed PRISMA guidelines and was registered in INPLASY. PubMed, Scopus, and EMBASE were searched from inception through January 2026 for observational studies evaluating the prognostic value of TAPSE/sPAP in adult patients with HF. Study selection, data extraction, and risk-of-bias assessment were performed independently by two reviewers. Owing to substantial heterogeneity, a qualitative synthesis with weighted pooled descriptive statistics was performed. **Results**: Fifteen observational studies including 5389 patients were analyzed, with a median follow-up of approximately 1.9 years, ranging from in-hospital outcomes to long-term follow-up of up to 15 years. Study populations encompassed a wide range of HF phenotypes and clinical settings, including acute and chronic HF, preserved and reduced ejection fraction, valvular heart disease, infiltrative cardiomyopathies, and advanced HF. Across studies, reduced TAPSE/sPAP was generally associated with adverse outcomes, including all-cause mortality and HF-related events, with reported hazard ratios ranging from approximately two- to five-fold. Prognostically relevant TAPSE/sPAP cut-off values tended to cluster within a relatively narrow range, with most thresholds between 0.36 and 0.40 and a weighted median of approximately 0.36. When reported, TAPSE/sPAP showed favorable discriminative performance for adverse outcomes. Overall methodological quality was predominantly fair. **Conclusions**: Across heterogeneous HF populations, impaired TAPSE/sPAP appears to be a consistent marker of adverse prognosis. These findings support TAPSE/sPAP as a practical, noninvasive indicator of RV–PA uncoupling that may contribute to risk stratification and phenotyping in heart failure. Prospective studies focusing on specific HF phenotypes are needed to clarify its role in longitudinal monitoring and therapeutic decision-making.

## 1. Introduction

Heart failure (HF) remains a leading cause of morbidity and mortality worldwide, despite major advances in pharmacological and device-based therapies [1,2]. Prognostic heterogeneity among patients with HF is substantial and not fully captured by conventional clinical and echocardiographic parameters, underscoring the need for robust markers that better reflect the underlying pathophysiology and enable refined risk stratification across different HF phenotypes and clinical settings [3,4,5].

In this context, increasing attention has been directed toward the right ventricle (RV) and its interaction with the pulmonary circulation [6]. Right ventricular dysfunction and pulmonary hypertension frequently coexist in HF and are powerful determinants of symptoms, exercise intolerance, and adverse outcomes [7,8,9]. However, isolated assessment of RV systolic function or pulmonary artery pressure provides only a partial representation of right heart performance, as RV contractility is intrinsically load dependent [10]. Consequently, parameters that fail to integrate RV function with its afterload may underestimate disease severity and prognostic risk [11].

The concept of right ventricular–pulmonary arterial (RV–PA) coupling has therefore emerged as a physiologically grounded framework to evaluate the ability of the RV to adapt to increased pulmonary vascular load [12,13]. Noninvasively, RV–PA coupling can be approximated using the ratio between tricuspid annular plane systolic excursion (TAPSE) and systolic pulmonary artery pressure (sPAP), a simple echocardiographic index that integrates RV longitudinal systolic performance with pulmonary afterload [14]. TAPSE/sPAP has been proposed as a surrogate of RV–PA interaction and has demonstrated prognostic relevance in selected populations, including pulmonary hypertension [15,16] and specific HF subgroups [17,18].

Over the past decade, multiple observational studies have investigated the prognostic significance of TAPSE/sPAP in HF. These studies span a broad spectrum of clinical scenarios, including acute and chronic HF, preserved and reduced ejection fraction, valvular heart disease, cardiac amyloidosis, advanced HF referred for transplantation, and post-heart transplant populations. However, the available evidence remains fragmented. Reported cut-off values associated with adverse outcomes vary across studies, study designs are heterogeneous, and patient populations differ markedly in terms of HF phenotype, disease severity, and clinical setting. As a result, the overall prognostic performance, reproducibility, and clinical applicability of TAPSE/sPAP across the HF continuum have not been systematically synthesized.

Moreover, it remains unclear whether TAPSE/sPAP provides consistent prognostic information beyond traditional RV or pulmonary pressure indices when applied across heterogeneous HF populations, and whether a clinically meaningful threshold can be identified to support bedside risk stratification. Addressing these gaps is essential to clarify the role of RV–PA coupling assessment in routine echocardiographic evaluation and to inform its potential integration into clinical decision-making.

Accordingly, the aim of the present systematic review was to comprehensively evaluate the prognostic value of the echocardiographic TAPSE/sPAP ratio in patients with heart failure. By synthesizing data from available observational studies, we sought to (i) characterize the clinical and echocardiographic profiles of patients in whom TAPSE/sPAP has been assessed, (ii) examine the consistency of its association with adverse outcomes across diverse HF phenotypes and settings, and (iii) identify convergent prognostic thresholds that may support its use as a practical and reproducible marker of RV–PA uncoupling in heart failure.

## 2. Materials and Methods

### 2.1. Study Design and Reporting Standards

This systematic review was conducted in accordance with the Preferred Reporting Items for Systematic Reviews and Meta-Analyses (PRISMA) guidelines [19]. The completed PRISMA checklist is included in the Supplementary Materials (Supplementary Materials S1).

The review protocol was prospectively registered in the INPLASY database [1,2,3,4,5,6,7,8,9,10,11,12,13,14,15,16,17,18] (registration number: INPLASY202620030; registration date: 8 February 2026; Supplementary Materials S2). Given the observational nature of the available evidence and the expected clinical and methodological heterogeneity across studies, a qualitative and descriptive synthesis was planned a priori, and no quantitative meta-analysis was undertaken.

### 2.2. Search Strategy

A comprehensive literature search was independently performed by two investigators (A.S. and G.F.G.) using the PubMed, Scopus, and EMBASE databases from inception through January 2026. The search strategy was designed to identify studies evaluating the prognostic role of RV–PA coupling assessed by echocardiography in patients with heart failure. The following terms and their combinations were used: “heart failure”, “acute heart failure”, “chronic heart failure”, “TAPSE”, “tricuspid annular plane systolic excursion”, “systolic pulmonary artery pressure”, “sPAP”, “TAPSE/sPAP”, “RV–PA coupling”, “right ventricular–pulmonary arterial coupling”, “prognosis”, “mortality”, and “outcomes”. The search was restricted to full-text articles published in English. This restriction was applied to ensure accurate methodological interpretation and consistent data extraction. Nevertheless, many studies conducted in non-English-speaking countries are published in English-language journals, and manual screening of reference lists did not identify additional eligible non-English publications containing extractable outcome data. Reference lists of eligible studies and relevant reviews were manually screened to identify additional pertinent publications.

### 2.3. Eligibility Criteria

Studies were eligible for inclusion if they enrolled adult patients with heart failure, regardless of phenotype or clinical setting, assessed RV–PA coupling using the echocardiographic TAPSE/sPAP ratio, and evaluated its association with prognostic outcomes such as all-cause mortality, cardiovascular mortality, heart failure hospitalization, or composite endpoints. Both prospective and retrospective observational cohort studies were considered eligible. Studies were excluded if they were non-clinical, experimental, or animal studies; case reports, small case series, conference abstracts, editorials, letters without original data, narrative reviews, or meta-analyses; studies assessing RV–PA coupling exclusively by cardiac magnetic resonance imaging or other non-echocardiographic modalities; if they did not report clinical outcomes; if echocardiographic data were insufficient to derive TAPSE/sPAP; or if they represented duplicate reports from the same study population, in which case the most complete or recent publication was retained.

### 2.4. Literature Screening and Data Extraction

Two investigators (A.S. and G.F.G.) independently screened all retrieved records by title and abstract, followed by full-text assessment of potentially eligible studies according to the predefined inclusion and exclusion criteria. Disagreements regarding study eligibility or data extraction were resolved through discussion and consensus, and when agreement could not be reached, a third reviewer was consulted for adjudication. Data extraction was independently performed by the same investigators using a predefined standardized form. Extracted data included study characteristics (author, year, country, design, sample size, and follow-up duration), patient demographics and heart failure phenotype, clinical variables and comorbidities, laboratory data, pharmacological treatment, echocardiographic parameters with particular focus on TAPSE, sPAP, and the TAPSE/sPAP ratio, as well as reported prognostic outcomes, effect estimates, and proposed TAPSE/sPAP cut-off values. A third investigator (G.L.N.) reviewed the extracted data to ensure accuracy and resolve any discrepancies.

### 2.5. Methodological Quality Assessment of Included Studies

The risk of bias of the included studies was independently assessed by two reviewers (A.S. and G.L.N.) using the National Institutes of Health (NIH) Quality Assessment Tool for Observational Cohort and Cross-Sectional Studies [20]. Each study was evaluated across the 14 methodological domains of the tool and classified as having good, fair, or poor methodological quality. Disagreements were resolved by consensus.

### 2.6. Statistical Analysis

Continuous and categorical variables extracted from the included studies were summarized using pooled descriptive statistics. Given the expected non-normal distribution of most clinical and echocardiographic parameters, central tendency was expressed as weighted medians with corresponding interquartile ranges. Study-level estimates were weighted by sample size to account for differences in cohort size and to limit the influence of small exploratory studies. When variables were reported exclusively as mean ± standard deviation, the reported mean value was retained for descriptive purposes, as transformation to medians was not feasible without access to individual-level data. Categorical variables were summarized as weighted proportions and expressed as weighted percentages.

Missing data were handled on a per-variable basis, with pooled estimates calculated only from studies reporting the relevant parameter and without imputation of missing values. For each variable, both the total sample size and the number of contributing studies were reported to ensure transparency regarding data availability and to facilitate interpretation of variability across studies.

Between-study heterogeneity was explored descriptively by systematically examining differences in patient characteristics, HF phenotype, clinical setting, study design, timing of TAPSE/sPAP assessment, and outcome definitions. Given the substantial clinical and methodological heterogeneity identified during the study design phase, a formal meta-analysis was considered methodologically inappropriate. Formal statistical measures of heterogeneity (e.g., Cochran’s Q or I^2^ statistics) were therefore not calculated, as no pooled effect estimates were derived. In accordance with current recommendations for systematic reviews of observational studies, no formal meta-analysis was performed. Although subgroup-specific quantitative synthesis (e.g., restricted to specific heart failure phenotypes such as HFrEF or acute heart failure) was considered during the study design phase, the limited number of studies within each phenotype, together with substantial clinical and methodological heterogeneity—including differences in clinical settings, TAPSE/sPAP measurement methods, cut-off values, follow-up duration, and outcome definitions—precluded reliable quantitative pooling even within phenotype-restricted subgroups. Therefore, a structured qualitative synthesis combined with pooled descriptive statistics was considered the most methodologically appropriate approach.

To complement the qualitative synthesis, descriptive graphical representations were constructed to facilitate visual comparison across studies. Specifically, a distribution plot of reported TAPSE/sPAP prognostic thresholds and a descriptive forest plot of reported effect estimates (hazard ratios or odds ratios) were generated to illustrate the convergence of cut-off values and the direction and magnitude of prognostic associations across studies. Because of heterogeneity in study design, endpoints, and statistical adjustment, these visualizations were intended for descriptive purposes only and were not used to derive pooled effect estimates.

Statistical processing was carried out with IBM SPSS Statistics (version 29.0; IBM Corp., Armonk, NY, USA). Statistical significance was defined as a two-tailed *p* value < 0.05.

### 2.7. Use of Artificial Intelligence Tools

Artificial intelligence (AI) tools were used solely to assist with language editing and improvement of grammar during manuscript preparation. Specifically, ChatGPT-5.3 (OpenAI, San Francisco, CA, USA) was used to review the text for spelling, grammar, and clarity. No AI tools were used for literature screening, data extraction, data analysis, or interpretation of results. All scientific content, interpretation of the findings, and final responsibility for the manuscript remain entirely with the authors.

## 3. Results

### 3.1. Selection of Studies Included in the Review

**Figure 1:**
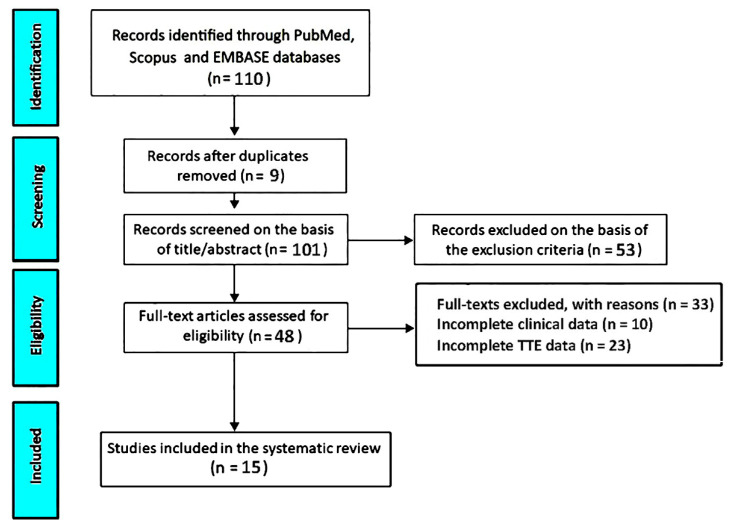
Schematic overview of the literature search, screening, and study selection steps following PRISMA standards. TTE, transthoracic echocardiography.

The systematic search strategy yielded a total of 110 publications. After removing nine duplicate records (8.2%), 101 unique studies (91.8%) were retained for the initial screening phase. Title and abstract review led to the exclusion of 53 articles (48.2%) that did not meet the predefined inclusion criteria. As a result, 48 full-text manuscripts (43.6%) were evaluated in detail for eligibility. Of these, 33 studies (30%) were excluded because of insufficient clinical information (n = 10, 9.1%) or incomplete transthoracic echocardiographic data (n = 23, 20.9%). In the final analysis, 15 studies (13.6%) [21,22,23,24,25,26,27,28,29,30,31,32,33,34,35] investigating the prognostic significance of TAPSE/sPAP in patients with HF were included in this systematic review (Figure 1).

### 3.2. Characteristics of Included Studies and Populations

The studies included in the present systematic review were published between 2013 and 2026 and were conducted across multiple geographic regions, including Europe, Asia, and South America. Specifically, contributing investigations originated from the Netherlands, Spain, Italy, the United Kingdom, Turkey, Romania, France, Germany, China, and Argentina, reflecting a broad international representation of HF populations with heterogeneous clinical profiles.

Most studies adopted an observational design, including both prospective and retrospective cohorts. The majority were monocentric investigations, while a smaller proportion were conducted across multiple centers. Sample sizes varied substantially, ranging from small exploratory cohorts with fewer than 50 patients to large observational studies exceeding 1500 participants, particularly in chronic HF registries. One seminal prospective study published in 2013 contributed early evidence on the prognostic relevance of TAPSE/sPAP in both reduced and preserved ejection fraction HF populations [21].

The included populations encompassed a wide spectrum of HF phenotypes and clinical settings. These ranged from acute and chronic HF cohorts with preserved, mildly reduced, or reduced ejection fraction to more specific populations, such as cardiac amyloidosis, HF secondary to severe aortic stenosis, degenerative or secondary mitral regurgitation, advanced HF referred for transplantation, and post–heart transplant recipients. In addition, one large contemporary study focused specifically on older patients hospitalized for acute heart failure with preserved ejection fraction (HFpEF), further expanding the representation of acute care settings and elderly populations [35]. Both ischemic and non-ischemic etiologies were represented, although several studies did not stratify outcomes according to HF etiology.

Across studies, RV–PA coupling was uniformly assessed using the echocardiographic TAPSE/sPAP ratio, although the timing of assessment (baseline, during hospitalization, or in the peri-procedural setting) and the statistical approach used to define prognostic thresholds varied. Reported TAPSE/sPAP cut-off values associated with adverse outcomes spanned a wide range, from 0.27 to 0.50 mm/mmHg, with a recurrent threshold around 0.36 mm/mmHg reported consistently across multiple cohorts, including both early and recent investigations.

A summary of study design, population characteristics, and reported TAPSE/sPAP thresholds is provided in Table 1.

### 3.3. Baseline Clinical and Laboratory Characteristics

A pooled descriptive summary of baseline clinical, laboratory, and pharmacological characteristics of the study population is reported in Table 2.

The pooled study population was characterized by an elderly predominance and a slight male majority, with most patients presenting with moderate functional limitation. Mean age consistently exceeded the mid-60s across studies, with a substantial proportion of cohorts composed of older adults, particularly in acute HF settings. Cardiovascular risk factors were highly prevalent, particularly hypertension, diabetes, dyslipidemia, and coronary artery disease, indicating a population with advanced cardiometabolic burden. Chronic obstructive pulmonary disease (COPD) and peripheral vascular disease were also frequently observed, highlighting the presence of significant systemic comorbidity.

Renal impairment was common, with reduced glomerular filtration rates and a substantial proportion of patients affected by chronic kidney disease. Hemoglobin levels suggested mild anemia in a considerable subset of the population, while natriuretic peptide concentrations were markedly elevated, reflecting advanced hemodynamic stress and HF severity. These features were consistently observed both in early foundational cohorts and in more recent studies focused on hospitalized elderly patients. Atrial fibrillation affected approximately one-quarter of patients, consistent with the high-risk clinical profile of the included cohorts.

Blood pressure values were generally within controlled ranges, while resting heart rate remained moderately elevated, consistent with chronic HF physiology. Regarding pharmacological treatment, most patients received guideline-directed medical therapy, including renin–angiotensin system inhibitors, beta-blockers, mineralocorticoid receptor antagonists, and loop diuretics. However, the pharmacological landscape varied substantially across studies, reflecting differences in enrollment period and clinical setting. In contrast, the use of newer disease-modifying therapies, such as angiotensin receptor–neprilysin inhibitors and sodium–glucose cotransporter 2 inhibitors, was relatively limited, reflecting the inclusion of earlier-generation study populations and real-world cohorts enrolled before the widespread adoption of contemporary HF therapies.

### 3.4. Echocardiographic Findings

A pooled summary of echocardiographic parameters across the included studies is presented in Table 3.

Across the included studies, RV–PA coupling, assessed by the TAPSE/sPAP ratio, was consistently impaired, indicating a widespread state of RV–PA uncoupling in patients with HF. This pattern was evident across both early landmark investigations and more contemporary cohorts, encompassing acute and chronic HF settings as well as preserved and reduced ejection fraction phenotypes. This finding reflects a reduced ability of the right ventricle to adapt to increased pulmonary vascular afterload and represents a key pathophysiological substrate underlying adverse clinical outcomes.

Notably, TAPSE/sPAP values tended to cluster within a relatively narrow pathological range across heterogeneous HF populations, suggesting a broadly consistent prognostic signal despite differences in study design, patient characteristics, and clinical settings. Despite substantial differences in patient age, clinical setting, and HF phenotype, pooled TAPSE/sPAP values remained consistently below established prognostic thresholds, reinforcing the notion of a shared pathophysiological mechanism of RV–PA uncoupling. The concomitant presence of elevated pulmonary artery pressures and reduced RV longitudinal systolic function further highlights the central role of afterload mismatch and RV contractile insufficiency in driving disease progression.

Additional echocardiographic features, including left atrial enlargement, elevated diastolic filling pressures, and frequent functional tricuspid regurgitation, provide supportive evidence of chronically increased left-sided pressures and secondary pulmonary hypertension, which mechanistically contribute to RV–PA uncoupling. These abnormalities were particularly prominent in studies enrolling elderly patients hospitalized for acute HF, underscoring the cumulative impact of chronic pressure overload and comorbidity burden on RV performance. Together, these structural and hemodynamic abnormalities create a substrate in which TAPSE/sPAP integrates both RV performance and pulmonary vascular load, explaining its superior prognostic discrimination compared with isolated RV or pulmonary pressure indices.

### 3.5. Prognostic Performance and Clinical Threshold of TAPSE/sPAP

In the included studies, the median follow-up duration was approximately 1.9 years, with a wide range spanning from in-hospital outcomes to very long-term follow-up of up to 15 years.

Most investigations assessed TAPSE/sPAP at a single baseline time point; however, one longitudinal cohort [23] provided repeated echocardiographic assessments over extended follow-up and demonstrated that temporal worsening of RV–PA coupling—characterized by increasing pulmonary pressures and declining TAPSE/sPAP—was associated with a higher risk of mortality. This finding suggests that changes in TAPSE/sPAP over time may carry additional prognostic information beyond baseline measurements, although such longitudinal analyses remain limited across the current evidence base.

Across 15 studies, the TAPSE/sPAP cut-offs associated with adverse outcomes tended to cluster within a relatively narrow range. The weighted pooled cut-off clustered around 0.36–0.38, with a weighted median of approximately 0.36, suggesting a broadly consistent prognostic signal across heterogeneous HF populations and clinical settings. Although individual studies reported optimal cut-offs ranging from 0.27 to 0.50, the majority identified clinically meaningful risk stratification within the 0.36–0.40 interval, reinforcing the reproducibility of TAPSE/sPAP as a marker of RV–PA uncoupling (Table 4).

However, the magnitude of the prognostic association varied across cohorts, and in some populations the predictive performance was more modest, highlighting the influence of clinical context, study design, and patient characteristics on the observed risk estimates.

When diagnostic accuracy metrics were reported, TAPSE/sPAP demonstrated good to excellent discriminative performance for adverse outcome prediction. Across studies providing ROC analyses, the AUC ranged from 0.65 to 0.89, with sensitivities generally between 85 and 90% and specificities between 70 and 80%, indicating a favorable balance between sensitivity and specificity for identifying high-risk patients. These findings suggest potential clinical usefulness of TAPSE/sPAP as a bedside parameter for prognostic stratification.

**Figure 2:**
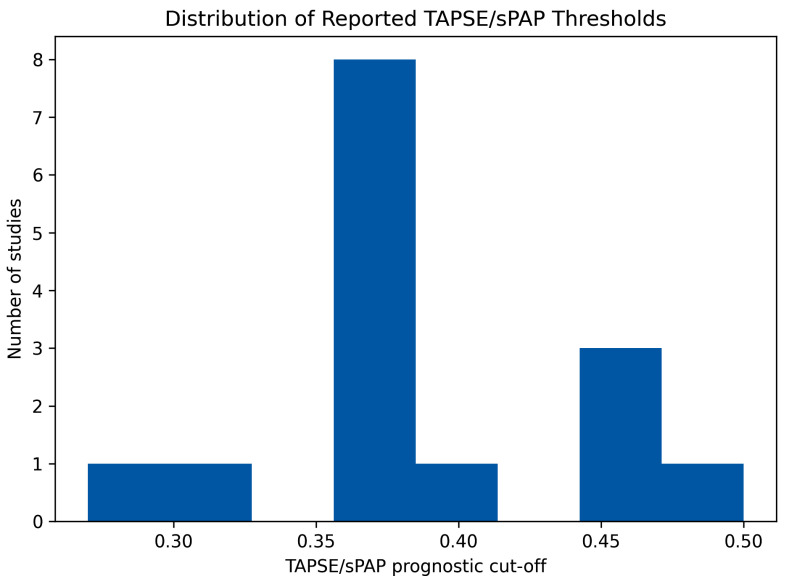
Histogram illustrating the distribution of reported TAPSE/sPAP thresholds associated with adverse outcomes in the 15 studies included in the systematic review.

To further illustrate the convergence of prognostic thresholds across studies, the distribution of study-specific TAPSE/sPAP cut-off values is shown in Figure 2.

Despite the overall reported range, most thresholds cluster around the 0.36–0.40 interval, visually supporting the presence of a recurrent prognostic threshold across heterogeneous heart failure populations.

**Figure 3:**
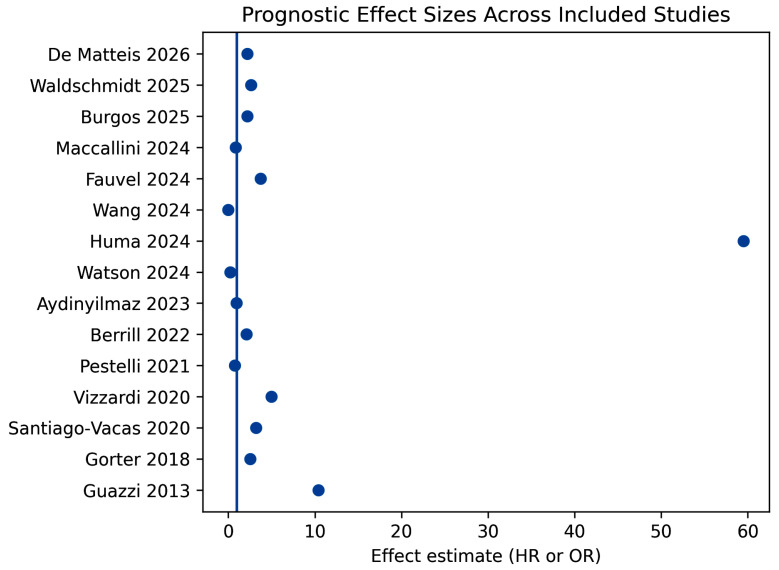
Forest plot summarizing the reported hazard ratios (HRs) or odds ratios (ORs) describing the association between impaired TAPSE/sPAP and adverse clinical outcomes in the included studies [21,22,23,24,25,26,27,28,29,30,31,32,33,34,35]. Effect estimates are presented as reported in the original studies and are not directly comparable because of differences in endpoint definitions, follow-up duration, and statistical adjustment. The plot illustrates the overall direction and magnitude of the prognostic associations across heterogeneous heart failure populations.

In addition, a descriptive forest plot summarizing the direction and magnitude of reported prognostic associations is presented in Figure 3.

Although effect estimates are heterogeneous and not directly comparable due to differences in endpoint definitions, statistical adjustment, and follow-up duration, the majority of studies demonstrate a consistent association between lower TAPSE/sPAP values and increased risk of adverse outcomes, supporting the overall prognostic relevance of RV–PA uncoupling in heart failure.

Despite substantial heterogeneity in study design, patient phenotype (acute vs. chronic HF, HFrEF vs. HFpEF, valvular and non-valvular HF), clinical setting, and endpoint definition, most included studies demonstrated an association between reduced TAPSE/sPAP and adverse outcomes. Lower TAPSE/sPAP values were associated with a two- to five-fold increase in the risk of mortality or HF-related events in most cohorts, while extreme risk estimates were observed in selected populations with advanced disease or short-term outcomes.

Collectively, these data support a graded association, whereby progressive RV–PA uncoupling, reflected by decreasing TAPSE/sPAP, is associated with worsening prognosis across the spectrum of HF.

To further explore potential sources of variability in the reported TAPSE/sPAP thresholds, an exploratory subgroup analysis was performed according to clinical setting and HF phenotype (Table 5).

In studies enrolling classical chronic HF populations (HFrEF or mixed HF cohorts), the optimal prognostic thresholds were highly consistent, clustering narrowly between 0.36 and 0.38, which largely explains the pooled cut-off of approximately 0.36 observed in the present analysis.

In contrast, acute HF cohorts showed greater variability in reported thresholds (0.27–0.40), likely reflecting the dynamic hemodynamic conditions and fluctuating pulmonary pressures typical of acute decompensation.

Finally, phenotype-specific cohorts, including HFpEF populations, valvular heart disease, transplant recipients, and subclinical outpatient cohorts, demonstrated broader and generally higher cut-off values (0.31–0.50). This variability may reflect differences in RV adaptation patterns, pulmonary vascular load, and disease stage across these populations.

Overall, these findings suggest that, while TAPSE/sPAP thresholds may vary according to clinical context, a value around ≈0.36 remains remarkably stable across the majority of classical HF cohorts, supporting its role as a clinically meaningful marker of RV–PA uncoupling.

**Figure 4:**
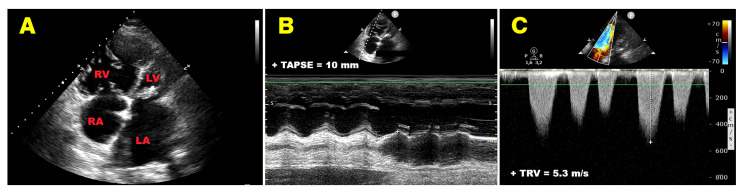
Representative example of severe right ventricular–pulmonary arterial (RV–PA) uncoupling in acute decompensated heart failure. (**A**) Transthoracic echocardiographic apical four-chamber view demonstrating marked right-sided chamber enlargement in a 60-year-old woman with severe rheumatic mitral stenosis and advanced congestive heart failure. (**B**) M-mode assessment of tricuspid annular plane systolic excursion (TAPSE), showing severely reduced right ventricular longitudinal systolic function (TAPSE = 10 mm). (**C**) Continuous-wave Doppler interrogation of the tricuspid regurgitant jet revealing markedly elevated tricuspid regurgitation velocity (TRV = 5.3 m/s), consistent with severe pulmonary hypertension. The combination of impaired right ventricular contractility and markedly increased pulmonary arterial afterload resulted in a profoundly reduced TAPSE/sPAP ratio (≈0.10 mm/mmHg), reflecting advanced RV–PA uncoupling and a high-risk hemodynamic profile.

As an illustrative example of severe RV–PA uncoupling, Figure 4 depicts a representative case of a 60-year-old woman hospitalized for acute decompensated HF in the setting of severe rheumatic mitral stenosis. The patient presented with advanced congestive heart failure, marked pulmonary hypertension, and severe functional tricuspid regurgitation (“tricuspidization” of the right ventricle). Transthoracic echocardiography demonstrated profoundly reduced RV longitudinal systolic function, with a TAPSE of 10 mm, in the presence of markedly elevated sPAP derived from a high-velocity tricuspid regurgitant jet (estimated tricuspid regurgitation velocity = 5.3 m/s). The resulting TAPSE/sPAP ratio was extremely reduced (≈0.10 mm/mmHg), reflecting advanced RV–PA uncoupling. This case exemplifies the pathophysiological scenario in which combined RV contractile impairment and severe pulmonary vascular afterload culminate in extreme reduction in TAPSE/sPAP, consistent with a high-risk hemodynamic profile and adverse prognosis.

### 3.6. Evaluation of Bias Across Included Studies

The risk of bias assessment of the included studies, performed using the NIH Quality Assessment Tool for Observational Cohort and Cross-Sectional Studies, is summarized in Table 6.

Overall methodological quality was predominantly fair, with fewer studies rated as good or poor, reflecting the typical profile of observational prognostic research in HF. Most studies clearly defined their research questions, populations, exposures, and outcomes, applied consistent statistical analyses, and had adequate follow-up, supporting the reliability of reported associations between TAPSE/sPAP and clinical outcomes. Importantly, exposure assessment and outcome ascertainment were generally well described across studies, as TAPSE/sPAP measurements were obtained using standardized echocardiographic methods, and clinical endpoints such as mortality or HF-related events were clearly defined.

However, common limitations included incomplete reporting of sample size justification, blinding procedures, repeated exposure assessment, and variable adjustment for confounders, resulting in frequent “not reported” ratings in several NIH domains. In particular, many studies did not report a formal sample size or power calculation, did not reassess TAPSE/sPAP over time (with exposure typically measured only at baseline), and provided limited details regarding assessor blinding or the handling of potential confounding factors in multivariable analyses. Two studies were classified as poor quality due to limited reporting and insufficient confounder adjustment, while the remainder demonstrated acceptable methodological rigor despite inherent observational design limitations.

**Figure 5:**
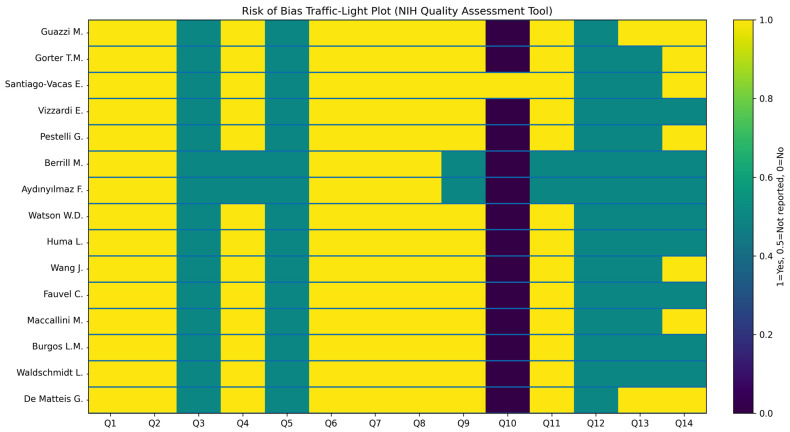
Study-level methodological quality assessment using the NIH Quality Assessment Tool for Observational Cohort and Cross-Sectional Studies [21,22,23,24,25,26,27,28,29,30,31,32,33,34,35]. Traffic-light plot illustrating the risk-of-bias assessment across the 14 methodological domains of the NIH tool for each included study. Yellow indicates “Yes”, teal indicates “Not Reported”, and purple indicates “No”. The figure highlights the domains most frequently affected by incomplete reporting, particularly sample size justification, blinding procedures, and repeated exposure assessment.

A study-level visualization of the methodological quality assessment across the 14 NIH domains is provided in Figure 5, illustrating the distribution of “Yes”, “No”, and “Not Reported” ratings for each study and highlighting the domains most frequently affected by incomplete reporting.

## 4. Discussion

### 4.1. Summary of Key Results

The present systematic review provides a comprehensive synthesis of the available evidence on the prognostic significance of RV–PA coupling, assessed by the echocardiographic TAPSE/sPAP ratio, across a broad spectrum of HF populations. The main finding is that impaired TAPSE/sPAP was consistently associated with adverse clinical outcomes, including all-cause mortality and HF-related events, across multiple HF phenotypes and clinical settings. Despite substantial heterogeneity in study design, patient characteristics, and outcome definitions, the overall body of evidence demonstrates a generally consistent prognostic signal, although the magnitude of the reported associations varied across studies. Notably, this association was observed across studies with markedly different follow-up durations, with a median follow-up of approximately 1.9 years and a range spanning from in-hospital outcomes to very long-term follow-up of up to 15 years, suggesting temporal stability of the prognostic relationship across diverse clinical scenarios.

Importantly, our analysis shows that prognostically relevant TAPSE/sPAP thresholds tended to cluster within a relatively narrow range, although variability between studies was present. Across 15 independent cohorts, the weighted pooled cut-off associated with adverse outcomes spanned from 0.27 to 0.50, with a clear concentration of thresholds between 0.36 and 0.40 and a weighted median of approximately 0.36. This pattern suggests the presence of a recurrent prognostic signal, while also highlighting that optimal thresholds may vary according to patient population, disease severity, and clinical context. This convergence across heterogeneous populations suggests that TAPSE/sPAP captures a fundamental pathophysiological process—namely RV–PA uncoupling—that transcends individual HF phenotypes and clinical contexts. Furthermore, when diagnostic performance was reported, TAPSE/sPAP demonstrated a favorable balance between sensitivity and specificity, indicating potential usefulness for clinical risk stratification, although the heterogeneity of study designs and outcome definitions should be considered when interpreting these findings.

Collectively, these findings indicate that TAPSE/sPAP provides prognostic information that is relatively consistent across observational studies and biologically plausible, supporting its role as an integrative echocardiographic marker of right heart function in HF. By simultaneously reflecting RV systolic performance and pulmonary vascular load, TAPSE/sPAP may offer incremental insight beyond isolated measures of RV function or pulmonary pressure, thereby providing a conceptual framework for right heart assessment across acute and chronic HF settings, as well as across the full spectrum of left ventricular ejection fraction.

### 4.2. Right Ventricular–Pulmonary Arterial Coupling: Pathophysiological Insights

The consistent prognostic performance of TAPSE/sPAP observed across heterogeneous HF populations can be explained by its strong pathophysiological grounding in the interaction between RV contractile function and pulmonary vascular load [36]. In HF, chronically elevated left-sided filling pressures are transmitted backward to the pulmonary circulation, leading to pulmonary venous hypertension [37,38] and, over time, to pulmonary vascular remodeling [39]. This progressive increase in pulmonary arterial afterload represents a major challenge for the right ventricle, whose thin-walled structure and limited contractile reserve make it particularly vulnerable to pressure overload [40,41]. In this context, the assessment of RV systolic function without accounting for afterload provides an incomplete and potentially misleading representation of right heart performance. TAPSE/sPAP addresses this limitation by integrating RV longitudinal systolic shortening with the pressure load against which the RV must eject, thereby serving as a noninvasive surrogate of RV–PA coupling [42]. It should be acknowledged, however, that TAPSE reflects predominantly the longitudinal component of RV systolic function and does not capture the radial contribution of the RV free wall and outflow tract. In clinical settings characterized by altered RV contraction patterns, alternative indices of RV–PA coupling based on other measures of RV systolic function—such as tissue Doppler-derived RV S’, fractional area change, or strain-based parameters normalized to pulmonary pressure—may provide complementary information [43,44].

A reduced TAPSE/sPAP ratio reflects a state in which RV contractility is insufficient relative to pulmonary arterial pressure, indicating an inability of the right ventricle to maintain effective ventriculo-arterial interaction [45]. This uncoupling marks the transition from adaptive RV remodeling to maladaptive failure, characterized by progressive RV dilation, reduced stroke volume, worsening functional tricuspid regurgitation, and systemic venous congestion. These changes can affect LV function through interventricular interdependence and promote multi-organ dysfunction, reduced exercise capacity, and increased vulnerability to adverse clinical events [45].

Importantly, the narrow pathological range of TAPSE/sPAP values observed across diverse HF phenotypes in the present review suggests that RV–PA uncoupling represents a common final pathway of disease progression, irrespective of left ventricular ejection fraction, etiology, or clinical setting. Whether in acute decompensation, chronic stable disease, valvular HF, or advanced HF referred for transplantation, a reduced TAPSE/sPAP consistently identified patients in whom right HF physiology played a central role in determining prognosis. This pathophysiological convergence provides a mechanistic explanation for the reproducibility of prognostic thresholds across studies and supports the biological plausibility of TAPSE/sPAP as an integrative marker of disease severity in HF.

### 4.3. Clinical Relevance and Practical Implications

The findings of this systematic review have several important implications for clinical practice and risk stratification in HF. TAPSE/sPAP is a simple, widely available, and reproducible echocardiographic parameter that can be obtained during routine transthoracic echocardiography without additional imaging time, contrast agents, or advanced post-processing. Because it relies on measurements already recommended in standard echocardiographic protocols, its systematic implementation does not require additional resources or specialized expertise. In contrast to more complex indices of RV function or invasive measures of pulmonary hemodynamics, TAPSE/sPAP offers an immediately accessible assessment of RV–PA interaction that can be readily incorporated into standard echocardiographic workflows.

It should be noted that calculation of the TAPSE/sPAP ratio requires a reliable estimation of sPAP from the tricuspid regurgitation Doppler signal, which may not be obtainable in all patients. When an adequate tricuspid regurgitation jet is absent or sPAP cannot be reliably estimated, the TAPSE/sPAP ratio cannot be calculated and should not be reported. In such situations, alternative approaches to assess RV–PA coupling may be considered, including indices incorporating other markers of RV systolic performance such as RV free-wall longitudinal strain normalized to pulmonary pressure (e.g., RV-GLS/sPAP), which have shown promising prognostic potential in recent studies [46,47].

The convergence of prognostic cut-off values around a narrow pathological range, most commonly between 0.36 and 0.40, across independent cohorts suggests that TAPSE/sPAP may serve as a clinically meaningful threshold to identify patients at increased risk of mortality or HF-related events across a broad range of HF phenotypes. Such a threshold could complement established risk markers by identifying patients in whom right heart dysfunction and pulmonary vascular disease are major contributors to clinical deterioration, even when left ventricular systolic function appears relatively preserved. In this regard, TAPSE/sPAP may be particularly valuable in phenotypes characterized by disproportionate right-sided involvement, such as HFpEF, valvular heart disease, and advanced heart failure, where traditional left-sided parameters may underestimate global disease severity [48].

Beyond baseline risk stratification, TAPSE/sPAP may also have a role in longitudinal patient assessment. Because both RV function and pulmonary arterial pressure are dynamic and potentially modifiable, changes in TAPSE/sPAP over time may reflect disease progression or response to therapy [49]. Serial assessment of RV–PA coupling may therefore provide incremental information beyond static measurements, supporting more refined follow-up strategies. Serial assessment of RV–PA coupling could therefore aid in monitoring treatment efficacy, guiding intensity of follow-up, and informing the timing of advanced therapies or referral to specialized centers [50]. In advanced HF settings, TAPSE/sPAP values in the ≈0.36–0.38 range have been associated with markedly worse outcomes in cohorts where urgent heart transplantation or mechanical circulatory support were included among clinical endpoints, suggesting that RV–PA uncoupling may help identify patients who warrant early evaluation in specialized HF centers. Conversely, in heart transplant recipients, higher postoperative TAPSE/sPAP values (e.g., >0.47) have been associated with improved short-term survival, supporting the potential role of RV–PA coupling assessment in the post-transplant setting. However, current evidence does not yet support a universally validated decision-trigger threshold for advanced therapy referral, and TAPSE/sPAP should be interpreted as part of a comprehensive clinical and hemodynamic assessment.

It should be acknowledged that most included studies reported sex distribution only at the cohort level and rarely provided sex-stratified analyses of TAPSE/sPAP prognostic performance or optimal thresholds. Given known sex-related differences in HF phenotypes, particularly the higher prevalence of HFpEF among women, the generalizability of pooled findings to female HF populations should therefore be interpreted with caution. Further investigation of potential sex-specific patterns of RV–PA coupling may help clarify whether prognostic thresholds or risk stratification strategies should differ between men and women.

Another important consideration is that several included cohorts were enrolled before the widespread implementation of contemporary guideline-directed medical therapy, including angiotensin receptor–neprilysin inhibitors and sodium–glucose cotransporter-2 inhibitors. These therapies may influence both left ventricular function and pulmonary hemodynamics, potentially improving RV–PA coupling. Consequently, the distribution of TAPSE/sPAP values and the proportion of patients meeting currently proposed high-risk thresholds could evolve in contemporary HF populations. Although TAPSE/sPAP reflects a fundamental pathophysiological interaction between RV contractility and pulmonary arterial afterload that is likely to remain clinically relevant, validation in cohorts treated with modern HF therapies will be important to determine whether currently proposed prognostic thresholds remain applicable or require recalibration.

Several alternative approaches have been proposed to evaluate RV–PA coupling, including invasive pressure–volume loop-derived indices such as the end-systolic elastance to arterial elastance ratio (Ees/Ea), as well as noninvasive surrogates derived from advanced imaging techniques such as three-dimensional echocardiography-based RV volumetric indices or parameters incorporating RV stroke volume [51]. Although these approaches may provide a more comprehensive characterization of RV–PA interaction, their clinical application is often limited by technical complexity, the need for specialized imaging protocols, or reliance on invasive measurements. In this context, TAPSE/sPAP represents a pragmatic and widely applicable surrogate of RV–PA coupling that can be readily implemented in routine echocardiographic evaluation while maintaining strong prognostic performance across diverse HF populations.

### 4.4. Practical Implementation and Future Research Directions

From a practical standpoint, TAPSE/sPAP can be calculated in most patients undergoing standard transthoracic echocardiography whenever both TAPSE and a measurable tricuspid regurgitation Doppler signal are available. While it may be reported as part of routine echocardiographic evaluation in HF, its clinical value is likely greatest in specific scenarios where right-sided hemodynamics may play a disproportionate role in symptoms or prognosis. These include patients with unexplained dyspnea or exercise intolerance, discordance between symptoms and left ventricular systolic function, suspected pulmonary hypertension, HFpEF with elevated filling pressures, significant valvular heart disease, or advanced HF being evaluated for escalation of therapy. In such settings, the assessment of TAPSE/sPAP may help identify RV–PA uncoupling and refine risk stratification beyond conventional left-sided parameters.

Based on the available evidence, a TAPSE/sPAP value below approximately 0.36 may be considered a clinically relevant “red flag” indicating significant RV–PA uncoupling and increased risk of adverse outcomes across multiple HF phenotypes. Nevertheless, interpretation should remain context-specific and integrated with the overall clinical and echocardiographic profile, as differences in HF phenotype, hemodynamic status, and comorbidity burden may influence the prognostic implications of this threshold.

Future prospective studies should aim to address several key questions, including whether serial changes in TAPSE/sPAP provide incremental prognostic information beyond baseline measurements, how contemporary guideline-directed medical therapy influences RV–PA coupling, and whether phenotype-specific thresholds may improve risk stratification. Particular attention should be given to HF subgroups in which right ventricular–pulmonary arterial interaction is likely to play a central pathophysiological role, including patients with HFpEF and secondary pulmonary hypertension, advanced HFrEF with right ventricular dysfunction, valvular heart disease associated with pulmonary hypertension (such as severe mitral or aortic valve disease), and patients being evaluated for advanced therapies such as mechanical circulatory support or heart transplantation. Adequately powered multicenter prospective cohorts with standardized echocardiographic acquisition and comprehensive clinical characterization will be essential to clarify these issues. In particular, studies designed with several hundred to a few thousand patients and longitudinal follow-up would be required to validate TAPSE/sPAP as a robust prognostic biomarker across different HF phenotypes.

Beyond its role as a prognostic marker, further investigation is needed to determine whether TAPSE/sPAP could serve as a therapeutic monitoring parameter or even a surrogate endpoint in clinical trials targeting right ventricular dysfunction or pulmonary vascular disease in HF populations. If validated in prospective interventional studies, RV–PA coupling indices such as TAPSE/sPAP may help identify treatment-responsive phenotypes and guide more individualized therapeutic strategies.

From an operational perspective, echocardiography laboratories may consider reporting TAPSE/sPAP whenever both TAPSE and Doppler-derived pulmonary pressure estimates are available, particularly in patients undergoing evaluation for HF, pulmonary hypertension, or unexplained dyspnea. Such integration into routine echocardiographic reporting may facilitate broader clinical familiarity with this parameter and promote its use in risk stratification and longitudinal patient assessment.

### 4.5. Prognostic Role of TAPSE/sPAP Beyond Heart Failure

The prognostic relevance of TAPSE/sPAP observed in HF is consistent with robust evidence derived from non-HF populations, in which RV–PA coupling plays a central role in disease progression and outcome determination. In pulmonary arterial hypertension (PAH), TAPSE/sPAP has been extensively validated as a noninvasive surrogate of RV–PA coupling and has consistently demonstrated strong associations with survival, exercise capacity, and clinical worsening [52,53,54]. In these patients, TAPSE/sPAP reflects the balance between RV contractile reserve and progressive pulmonary vascular remodeling, outperforming isolated measures of RV function or pulmonary artery pressure in prognostic discrimination.

Similarly, in systemic sclerosis and interstitial lung diseases, including idiopathic pulmonary fibrosis, TAPSE/sPAP has emerged as a sensitive marker of early RV dysfunction and adverse prognosis, even in the absence of overt pulmonary hypertension at rest [55,56,57,58]. In these settings, the subclinical impairment of RV–PA interaction precedes overt right HF and is associated with reduced functional capacity and increased mortality. The ability of TAPSE/sPAP to detect early RV–PA uncoupling in diseases primarily driven by pulmonary vascular or parenchymal pathology underscores its pathophysiological robustness and broad applicability.

Taken together, evidence from PAH and pulmonary disease populations supports the concept that RV–PA uncoupling represents a final common pathway leading to adverse outcomes across a wide range of cardiovascular and pulmonary conditions. The consistency of TAPSE/sPAP as a prognostic marker in both HF and non-HF populations reinforces its biological validity and suggests that its prognostic power is not disease-specific but rather reflects a fundamental mechanism of right HF. This cross-disease consistency further strengthens the rationale for TAPSE/sPAP as an integrative and generalizable marker of risk in HF, situating RV–PA coupling within a broader framework of cardio-pulmonary interaction.

### 4.6. Limitations of the Available Evidence

Several limitations of the studies included in this systematic review should be acknowledged when interpreting the present findings. First, all included investigations were observational in nature, predominantly monocentric, and based on either prospective or retrospective cohort designs. As such, the possibility of residual confounding cannot be excluded, and causal inferences regarding the relationship between TAPSE/sPAP and clinical outcomes cannot be definitively established. Adjustment for potential confounders varied across studies, with differences in the selection of covariates and statistical models and, in some cases, limited multivariable adjustment, which may have influenced the magnitude of the reported associations.

Second, the overall methodological quality of the included studies was judged to be predominantly fair, with only a minority rated as good and two classified as poor according to the NIH Quality Assessment Tool. Several key methodological domains were frequently rated as “not reported,” including sample size justification, blinding of outcome assessors, and repeated assessment of exposure over time. In particular, TAPSE/sPAP was generally measured at a single time point, precluding the evaluation of temporal changes in RV–PA coupling and their potential prognostic implications. As a result, the ability to assess TAPSE/sPAP as a dynamic marker of disease progression or treatment response remains limited.

Third, substantial clinical and methodological heterogeneity was present across studies. Included populations spanned a wide range of HF phenotypes, disease severities, and clinical settings, including acute and chronic HF, preserved and reduced ejection fraction, valvular heart disease, infiltrative cardiomyopathies, and advanced HF referred for transplantation. In addition, the timing of echocardiographic assessment, methods used to define TAPSE/sPAP cut-off values, and selected prognostic endpoints varied considerably. Although this heterogeneity limits direct comparability between studies and reduces the feasibility of formal quantitative pooling, it should be considered when interpreting the pooled descriptive findings. At the same time, this diversity reflects real-world clinical practice and supports the broad applicability of the observed prognostic associations.

Another limitation relates to the long time span covered by the included studies (2013–2026), during which HF management evolved substantially with the introduction of contemporary guideline-directed medical therapy, including angiotensin receptor–neprilysin inhibitors and sodium–glucose cotransporter-2 inhibitors. Although an exploratory comparison across publication periods did not reveal a clear temporal shift in the prognostic thresholds reported for TAPSE/sPAP—most of which remained clustered around 0.36–0.40—the interpretation of potential temporal trends is limited by heterogeneity in study populations and by the non-uniform reporting of background medical therapy. Moreover, several recently published cohorts included patients enrolled before the widespread implementation of these therapies, further limiting the ability to evaluate the influence of modern treatment on the prognostic performance of TAPSE/sPAP.

Although TAPSE and Doppler-derived sPAP are widely used parameters recommended in standard echocardiographic protocols, complete methodological standardization across the included studies was not always present. Differences in image acquisition protocols, estimation of right atrial pressure, and timing of echocardiographic assessment may have contributed to inter-study variability in TAPSE/sPAP measurements. In addition, reporting of interobserver and intraobserver variability for echocardiographic measurements was inconsistent across studies, and reproducibility analyses were not uniformly available, which may represent an additional source of methodological heterogeneity. Across the included studies, the feasibility of calculating TAPSE/sPAP was not systematically reported; however, when described, Doppler estimation of sPAP—and therefore calculation of TAPSE/sPAP—was feasible in approximately 60–90% of patients. This variability reflects the dependence of Doppler-based pulmonary pressure estimation on the presence of an adequate tricuspid regurgitation signal and overall image quality in routine echocardiographic practice.

In addition, atrial fibrillation was relatively common across the included cohorts, yet rhythm-specific validation of TAPSE/sPAP (including atrial fibrillation-specific thresholds or interaction analyses) was rarely reported. Given that irregular rhythm and beat-to-beat variability may influence both TAPSE measurement and Doppler-based estimation of pulmonary pressures, the prognostic performance of TAPSE/sPAP in patients with atrial fibrillation remains less well characterized and should therefore be interpreted with appropriate caution.

Finally, the echocardiographic estimation of sPAP is subject to well-recognized technical and physiological limitations that may affect the accuracy of the TAPSE/sPAP ratio. In patients with right HF and severe tricuspid regurgitation, a marked enlargement of the regurgitant orifice may lead to reduced tricuspid regurgitation velocity and truncation of the continuous-wave Doppler signal, resulting in an underestimation of sPAP [59]. Even mild tricuspid regurgitation may produce an incomplete Doppler envelope, particularly in patients with COPD or advanced lung disease, further compromising sPAP assessment [60]. In addition, intrathoracic pressure variations and respiratory dynamics can influence transtricuspid pressure gradient measurements [61,62]. Estimation of right atrial pressure based on inferior vena cava size and collapsibility is also imperfect and represents a major source of error, with potential overestimation propagating into sPAP calculation [63,64,65].

Nevertheless, despite these limitations, the consistency of the association between reduced TAPSE/sPAP and adverse outcomes across studies of varying quality, design, and clinical context strengthens the robustness and clinical relevance of the overall findings.

## 5. Conclusions

This systematic review suggests that the echocardiographic TAPSE/sPAP ratio is a promising and reproducible marker of adverse prognosis across a wide spectrum of HF populations. Despite substantial heterogeneity in study design, clinical setting, and HF phenotype, reduced TAPSE/sPAP was generally associated with increased mortality and HF-related events. This overall consistency of the observed associations across acute and chronic settings, as well as across preserved and reduced ejection fraction phenotypes, supports the potential generalizability of TAPSE/sPAP as a prognostic indicator.

The convergence of prognostic thresholds around a relatively narrow range (approximately 0.36–0.40) may reflect a common and clinically meaningful pathophysiological pathway related to RV–PA uncoupling in HF progression. By integrating RV systolic performance with pulmonary vascular load, TAPSE/sPAP captures a key mechanism underlying right heart dysfunction that is not fully reflected by isolated RV or pulmonary pressure measures.

Given its simplicity, wide availability, and strong biological rationale, TAPSE/sPAP may represent a useful adjunct to routine echocardiographic assessment for risk stratification and patient phenotyping in HF. However, current evidence remains primarily observational, and its incorporation into standard echocardiographic evaluation should be interpreted as complementary to existing clinical and imaging parameters rather than as a stand-alone decision tool. Prospective studies focusing on specific HF phenotypes are warranted to define its role in longitudinal monitoring and therapeutic decision-making and to determine whether TAPSE/sPAP-guided strategies can meaningfully improve clinical outcomes in HF.

## Figures and Tables

**Table 1:** Summary of study design, sample size, demographic characteristics, heart failure phenotype, and TAPSE/sPAP cut-off values across the 15 studies included in the systematic review [21,22,23,24,25,26,27,28,29,30,31,32,33,34,35].

Study Name, Publication Year and Country	Design	Size (n)	Mean Age (% Males)	HF Population	TAPSE/sPAP Cut-Off
Guazzi M. (2013), Italy [21]	Prospective, Monocentric	293	63 (79)	HFrEF cohort, predominantly ischemic	0.36
Gorter T.M. (2018), The Netherlands [22]	Prospective, Monocentric	97	73.7 (31)	HFpEF cohort, predominantly non-ischemic	0.36
Santiago-Vacas E. (2020), Spain [23]	Prospective, monocentric	1557	67 (69.9)	Mixed HF cohort, predominantly ischemic	0.36
Vizzardi E. (2020), Italy [24]	Retrospective, monocentric	56	81.6 (42.9)	HF secondary to severe aortic stenosis	0.5
Pestelli G. (2021), Italy [25]	Retrospective, monocentric	200	79 (50)	Mixed HF population, predominantly non-ischemic	0.36
Berrill M. (2022), United Kingdom [26]	Prospective, monocentric	418	78.7 (53.1)	Acute HF, mixed etiology	0.27
Aydınyılmaz F. (2023), Turkey [27]	Prospective, monocentric	245	64 (75.1)	Outpatient cohort with subclinical/asymptomatic HF	0.47
Watson W.D. (2024), United Kingdom [28]	Prospective, monocentric	456	NR (75)	Advanced HFrEF with secondary mitral regurgitation	0.37
Huma L. (2024), Romania [29]	Prospective, monocentric	46	45.2 (86.9)	Heart transplant recipients	0.47
Wang J. (2024), China [30]	Retrospective, monocentric	414	74.9 (41.1)	HFpEF	0.45
Fauvel C. (2024), France [31]	Prospective, multicentric	322	68 (70)	Acute HF, mixed etiology	0.4
Maccallini M. (2024), Spain [32]	Prospective, multicentric	233	80.4 (73)	HF due to cardiac amyloidosis	0.38
Burgos L.M. (2025), Argentina [33]	Retrospective, monocentric	361	77 (49)	Acute HFpEF/HFmrEF, mixed etiology	0.38
Waldschmidt L. (2025), Germany [34]	Retrospective, monocentric	293	81 (49.1)	HF associated with degenerative mitral regurgitation	0.31
De Matteis G. (2026), Italy [35]	Retrospective, monocentric	398	81.7 (44)	Acute HF, mixed etiology	0.36

Studies are ordered by year of publication. HF phenotypes include acute and chronic heart failure, preserved and reduced ejection fraction, valvular heart disease, infiltrative cardiomyopathies, advanced heart failure, and post-heart transplant populations. HF, heart failure; HFmrEF, heart failure with mildly reduced ejection fraction; HFpEF, heart failure with preserved ejection fraction; HFrEF, heart failure with reduced ejection fraction; NR, not reported; sPAP, systolic pulmonary artery pressure; TAPSE, tricuspid annular plane systolic excursion.

**Table 2:** Weighted pooled descriptive summary of baseline clinical, laboratory, and treatment characteristics across included studies.

Parameter	Weighted Median	Weighted IQR (Q1–Q3)	Studies Included	Total N
Mean age (years)	68.21	65.25–78.48	14	4933
%males	58.04	49.01–69.98	15	5389
BSA (m^2^)	1.84	1.84–1.84	2	246
BMI (Kg/m^2^)	25.92	24.84–27.24	9	2071
NYHA	2.25	2.21–2.81	11	4242
Hypertension (%)	65.42	63.61–66.08	9	3485
Diabetes (%)	28.75	22.46–35.95	10	3883
Dyslipidemia (%)	53.84	48.00–58.37	3	916
Smoking (%)	33.80	28.80–49.40	2	555
CAD (%)	46.06	26.73–46.36	13	4678
Peripheral vasculopathy (%)	13.30	13.30–13.30	1	1557
COPD (%)	16.08	13.08–16.18	8	3147
Creatinine (mg/dL)	1.17	0.95–1.32	4	1198
eGFR (mL/min/m^2^)	59.40	55.71–61.15	4	2139
CKD/ESRD (%)	17.44	6.58–23.10	6	1940
PMK/ICD (%)	15.08	9.51–15.24	4	2510
Hb (g/dL)	12.35	11.94–12.73	6	2811
NT-proBNP (pg/mL)	5272.18	2571.50–6255.60	10	4263
AF (%)	24.49	22.48–34.27	8	3582
HR (bpm)	75.00	72.81–80.88	6	1543
SBP (mmHg)	129.92	124.70–133.12	7	1551
DBP (mmHg)	73.77	71.31–79.70	7	1551
Previous HF Hospital. (%)	34.00	34.00–34.00	1	97
Hospital Length of Stay (days)	9.79	9.16–10.32	4	1058
Antiplatelets (%)	18.13	16.70–46.62	2	478
Anticoagulants (%)	14.30	14.30–34.95	2	478
ACEi/ARB (%)	76.47	55.12–82.43	9	4118
BB (%)	85.67	51.26–87.94	8	3720
Amiodarone (%)	6.50	6.50–6.50	1	245
ARNI (%)	7.33	6.22–8.43	4	2749
MRA (%)	54.70	42.34–63.26	7	3459
Ivabradine (%)	18.70	17.94–19.45	2	1802
Furosemide (%)	84.79	78.61–88.53	8	3559
SGLT2i (%)	0.29	0.00–1.97	3	800
Digoxin (%)	42.40	42.40–42.40	1	1557
Nitrates (%)	50.90	50.90–50.90	1	1557
Statins (%)	48.20	48.20–51.71	2	478

Values are reported as weighted medians and interquartile ranges (IQRs), weighted by study sample size. Central values were derived from mean ± standard deviation when medians were not available. The number of studies and total number of patients contributing to each variable are reported. ACEi, angiotensin-converting enzyme inhibitors; AF, atrial fibrillation; ARB, angiotensin receptor blockers; ARNI, angiotensin receptor–neprilysin inhibitor; BB, beta-blockers; BMI, body mass index; BSA, body surface area; CAD, coronary artery disease; CKD, chronic kidney disease; COPD, chronic obstructive pulmonary disease; DBP, diastolic blood pressure; eGFR, estimated glomerular filtration rate; ESRD, end-stage renal disease; Hb, hemoglobin; HF, heart failure; Hospital., hospitalization; HR, heart rate; IQR, interquartile range; MRA, mineralocorticoid receptor antagonists; NT-proBNP, N-terminal pro–B-type natriuretic peptide; NYHA, New York Heart Association; SBP, systolic blood pressure; SGLT2i, sodium–glucose cotransporter 2 inhibitors.

**Table 3:** Weighted pooled descriptive summary of echocardiographic parameters across included studies.

Parameter	Weighted Median	Weighted IQR (Q1–Q3)	Studies Included (n)	Total N
Cardiac index (L/min/m^2^)	3.05	2.98–3.13	2	419
E/e’	12.06	11.42–12.52	6	1555
IVS (mm)	15.18	14.22–16.14	2	289
PW (mm)	15.00	15.00–15.00	1	56
LAVi (mL/m^2^)	51.59	48.53–53.63	3	695
LVEDD (mm)	57.41	55.55–59.29	5	2712
LVESD (mm)	46.59	40.37–46.95	3	2200
LVMi (g/m^2^)	103.16	96.00–111.10	3	353
LVEDV (mL)	115.73	106.30–121.56	4	871
LVESV (mL)	48.80	48.80–55.54	3	549
LVEF (%)	34.90	34.16–52.52	13	4610
Moderate-to-severe MS (%)	3.30	3.30–3.30	1	414
Moderate-to-severe AR (%)	2.20	2.20–4.77	3	670
Moderate-to-severe MR (%)	27.51	12.77–32.79	7	3075
Moderate-to-severe AS (%)	2.70	2.70–6.83	3	703
Moderate-to-severe TR (%)	34.19	27.90–36.48	4	903
RV-FAC (%)	31.00	31.00–36.72	3	353
RV-GLS (%)	17.60	17.60–17.60	1	56
RV-GLS/sPAP (%/mmHg)	0.48	0.48–0.48	1	56
RV-S’ (cm/s)	10.09	9.24–10.30	3	398
RVIT (mm)	27.00	27.00–30.14	3	699
TAPSE (mm)	17.70	17.70–18.35	14	4971
sPAP (mmHg)	44.05	39.61–45.33	14	4971
TAPSE/sPAP (mm/mmHg)	0.40	0.38–0.46	15	4698

Values are reported as weighted medians and interquartile ranges (IQRs), weighted by study sample size. Missing values were excluded on a per-parameter basis. Parameters reflect left- and right-sided cardiac structure, function, and pulmonary hemodynamics. AR, aortic regurgitation; AS, aortic stenosis; E/e’, ratio of early mitral inflow velocity to mitral annular early diastolic velocity; IQR, interquartile range; IVS, interventricular septum thickness; LAVi, left atrial volume index; LVEDD, left ventricular end-diastolic diameter; LVEDV, left ventricular end-diastolic volume; LVEF, left ventricular ejection fraction; LVESD, left ventricular end-systolic diameter; LVESV, left ventricular end-systolic volume; LVMi, left ventricular mass index; MR, mitral regurgitation; MS, mitral stenosis; RV-FAC, right ventricular fractional area change; RV-GLS, right ventricular global longitudinal strain; RV-S’, right ventricular systolic velocity; sPAP, systolic pulmonary artery pressure; TAPSE, tricuspid annular plane systolic excursion; TR, tricuspid regurgitation.

**Table 4:** Prognostic cut-off values and clinical outcomes associated with TAPSE/sPAP across included studies [21,22,23,24,25,26,27,28,29,30,31,32,33,34,35].

Study	TAPSE/sPAP (mm/mmHg)	Cut-Off	Outcome	F.U. (Years)	Risk Estimate
Guazzi M. [21]	0.50 ± 0.18	0.36	All-cause mortality	1.7	HR 10.4
Gorter T.M. [22]	0.44 ± 0.20	0.36	Pre-capillary pulmonary hypertension	NR	HR 2.51
Santiago-Vacas E. [23]	0.41 ± 0.17	0.36	Mortality and HF-related hospitalizations	15	HR 3.18
Vizzardi E. [24]	0.50 ± 0.23	0.50	Composite of death and HF hospitalization	10	HR 4.98
Pestelli G. [25]	0.38 ± 0.16	0.36	HF-related mortality	2.7	HR 0.74
Berrill M. [26]	0.30 ± 0.13	0.27	Worse clinical outcome	2	HR 2.12
Aydınyılmaz F. [27]	0.58 ± 0.17	0.47	HF hospitalization	0	HR 0.94
Watson W.D. [28]	0.35 ± 0.19	0.37	Death, urgent HTx or MCS	2.39	HR 0.21
Huma L. [29]	0.56 ± 0.19	0.47	6-month survival	0.5	OR 59.5
Wang J. [30]	0.64 ± 0.29	0.45	All-cause mortality	5	HR 0.006
Fauvel C. [31]	0.50 ± 0.26	0.40	In-hospital MACEs	0	OR 3.75
Maccallini M. [32]	0.48 ± 0.24	0.38	Death or HF hospitalization	1.86	HR 0.86
Burgos L.M. [33]	0.41 ± 0.21	0.38	Long-term all-cause mortality	1.74	HR 2.21
Waldschmidt L. [34]	0.43 ± 0.20	0.31	2-year mortality	2	HR 2.64
De Matteis G. [35]	0.50 ± 0.20	0.36	In-hospital all-cause mortality	0	HR 2.18

Mean TAPSE/sPAP values, study-specific optimal cut-off thresholds, clinical endpoints, and corresponding risk estimates as reported in the original studies. Hazard ratios (HRs) and odds ratios (ORs) are presented according to the primary prognostic outcome of each study and are not directly comparable due to differences in endpoint definition, follow-up duration, and statistical adjustment. Follow-up is reported in years; in-hospital outcomes are coded as 0. F.U., follow-up; HF, heart failure; HR, hazard ratio; HTx, heart transplantation; MCS, mechanical circulatory support; MACEs, major adverse cardiovascular events; NR, not reported; OR, odds ratio; sPAP, systolic pulmonary artery pressure; TAPSE, tricuspid annular plane systolic excursion.

**Table 5:** Exploratory subgroup analysis of TAPSE/sPAP prognostic cut-offs according to clinical setting and heart failure phenotype [21,22,23,24,25,26,27,28,29,30,31,32,33,34,35].

Clinical Subgroup	Reported TAPSE/sPAP Cut-Off Range	Interpretation
Chronic HF cohorts (HFrEF or mixed HF) [21,23,25,28,32]	0.36–0.38	Highly consistent thresholds across classical HF populations; drives pooled cut-off ≈ 0.36
Acute HF cohorts [26,31,33,35]	0.27–0.40	Broader variability likely reflecting dynamic hemodynamic conditions during acute decompensation
Phenotype-specific populations (HFpEF, valvular HF, transplant, subclinical HF) [22,24,27,29,30,34]	0.31–0.50	Higher or more variable thresholds possibly reflecting different RV adaptation patterns and pulmonary vascular load

HF, heart failure; HFpEF, heart failure with preserved ejection fraction; HFrEF, heart failure with reduced ejection fraction; RV, right ventricular; sPAP, systolic pulmonary artery pressure; TAPSE, tricuspid annular plane systolic excursion.

**Table 6:** Risk of bias assessment of the included studies [21,22,23,24,25,26,27,28,29,30,31,32,33,34,35] performed using the NIH Quality Assessment Tool for Observational Cohort and Cross-Sectional Studies.

Study Name	Q1	Q2	Q3	Q4	Q5	Q6	Q7	Q8	Q9	Q10	Q11	Q12	Q13	Q14	Yes (n)	Overall
Guazzi M. [21]	Y	Y	NR	Y	NR	Y	Y	Y	Y	N	Y	NR	Y	Y	10	Good
Gorter T.M. [22]	Y	Y	NR	Y	NR	Y	Y	Y	Y	N	Y	NR	NR	Y	9	Fair
Santiago-Vacas E. [23]	Y	Y	NR	Y	NR	Y	Y	Y	Y	Y	Y	NR	NR	Y	10	Good
Vizzardi E. [24]	Y	Y	NR	Y	NR	Y	Y	Y	Y	N	Y	NR	NR	NR	8	Fair
Pestelli G. [25]	Y	Y	NR	Y	NR	Y	Y	Y	Y	N	Y	NR	NR	Y	9	Fair
Berrill M. [26]	Y	Y	NR	NR	NR	Y	Y	Y	NR	N	NR	NR	NR	NR	5	Poor
Aydınyılmaz F. [27]	Y	Y	NR	NR	NR	Y	Y	Y	NR	N	NR	NR	NR	NR	5	Poor
Watson W.D. [28]	Y	Y	NR	Y	NR	Y	Y	Y	Y	N	Y	NR	NR	NR	8	Fair
Huma L. [29]	Y	Y	NR	Y	NR	Y	Y	Y	Y	N	Y	NR	NR	NR	8	Fair
Wang J. [30]	Y	Y	NR	Y	NR	Y	Y	Y	Y	N	Y	NR	NR	Y	9	Fair
Fauvel C. [31]	Y	Y	NR	Y	NR	Y	Y	Y	Y	N	Y	NR	NR	NR	8	Fair
Maccallini M. [32]	Y	Y	NR	Y	NR	Y	Y	Y	Y	N	Y	NR	NR	Y	9	Fair
Burgos L.M. [33]	Y	Y	NR	Y	NR	Y	Y	Y	Y	N	Y	NR	NR	NR	8	Fair
Waldschmidt L. [34]	Y	Y	NR	Y	NR	Y	Y	Y	Y	N	Y	NR	NR	NR	8	Fair
De Matteis G. [35]	Y	Y	NR	Y	NR	Y	Y	Y	Y	N	Y	NR	Y	Y	10	Good

Each item was rated as Yes (Y), No (N), Not Reported (NR), Not Applicable (NA), or Cannot Determine (CD). Overall study quality (Good, Fair, or Poor) was derived from the total number of items rated as “Yes,” according to prespecified criteria. CD, cannot determine; NA, not applicable; NIH, National Institutes of Health; NR, not reported.

## Data Availability

Data extracted from included studies will be publicly available on Zenodo (18 February 2026) (https://zenodo.org).

## Supplementary Materials

The following supporting information can be downloaded at: https://www.mdpi.com/article/10.3390/jcm15062334/s1, Supplementary Materials S1: PRISMA 2020 checklist; Supplementary Materials S2: The full INPLASY record [1,2,3,4,5,6,7,8,9,10,11,12,13,14,15,16,17,18] (https://inplasy.com/inplasy-2026-2-0030/, accessed on 8 February 2026).
