# Supplementary material for: Echocardiographic Assessment of Right Ventricular–Pulmonary Arterial Coupling in Heart Failure: Prognostic Insights from a Systematic Review
Source: J Clin Med. 2026 Mar 18;15(6):2334. doi: 10.3390/jcm15062334 (PMC13026980; doi:10.3390/jcm15062334)
Supplement: Supplementary file 1 [file jcm-15-02334-s001.zip › Supplementary Materials S2.pdf]

Sonaglioni et al. INPLASY protocol 202620030. doi:10.37766/inplasy2026.2.0030

Downloaded from https://inplasy.com/inplasy-2026-2-0030/

International Platform of Registered Systematic Review and Meta-analysis Protocols

INPLASY

Echocardiographic Assessment of Right Ventricular–Pulmonary Arterial Coupling in Heart Failure: Prognostic Insights from a Systematic Review

Sonaglioni, A; Gramaglia, GF; Baravelli, M; Lombardo, M; Nicolosi, GL; Harari, S.

ADMINISTRATIVE INFORMATION

Support - Ministero della Salute Ricerca Corrente.

Review Stage at time of this submission - Completed but not published.

Conflicts of interest - None declared.

INPLASY registration number: INPLASY202620030

Amendments - This protocol was registered with the International Platform of Registered Systematic Review and Meta-Analysis Protocols (INPLASY) on 8 February 2026 and was last updated on 8 February 2026.

Corresponding author: Andrea Sonaglioni; sonaglioniandrea@gmail.com; Author Affiliation: IRCCS MultiMedica.

INPLASY202620030

doi: 10.37766/inplasy2026.2.0030

Received: 8 February 2026

Published: 8 February 2026

INTRODUCTION

Review question / Objective The aim of the present systematic review was to comprehensively evaluate the prognostic value of the echocardiographic tricuspid annular plane systolic excursion (TAPSE)/systolic pulmonary artery pressure (sPAP) ratio in patients with heart failure (HF). By synthesizing data from available observational studies, we sought to (i) characterize the clinical and echocardiographic profiles of patients in whom TAPSE/sPAP has been assessed, (ii) examine the consistency of its association with adverse outcomes across diverse HF phenotypes and settings, and (iii) identify convergent prognostic thresholds that may support its use as a practical and reproducible marker of RV–PA uncoupling in heart failure.

Rationale Over the past decade, multiple observational studies have investigated the prognostic significance of TAPSE/sPAP in HF. These studies span a broad spectrum of clinical scenarios, including acute and chronic HF, preserved and reduced ejection fraction, valvular heart disease, cardiac amyloidosis, advanced HF referred for transplantation, and post–heart transplant populations. However, the available evidence remains fragmented. Reported cut-off values associated with adverse outcomes vary across studies, study designs are heterogeneous, and patient populations differ markedly in terms of HF phenotype, disease severity, and clinical setting. As a result, the overall prognostic performance, reproducibility, and clinical applicability of TAPSE/sPAP across the HF continuum have not been systematically synthesized. Moreover, it remains unclear whether TAPSE/sPAP provides consistent prognostic information beyond traditional RV or pulmonary pressure indices when applied across heterogeneous HF populations, and whether a clinically meaningful threshold can be identified to support bedside risk stratification. Addressing these gaps is essential to clarify the role of RV–PA coupling assessment in routine echocardiographic evaluation and to inform its potential integration into clinical decision-making.

Condition being studied Heart failure (HF) remains a leading cause of morbidity and mortality wor ldwide , de spi t e ma jor adv anc e s i n pharmacological and device-based therapies [1,2] Prognostic heterogeneity among patients with HF is substantial and not fully captured by conventional clinical and echocardiographic parameters, underscoring the need for robust markers that better reflect the underlying pathophysiology and enable refined risk stratification across different HF phenotypes and clinical settings [3-5]. In this context, increasing attention has been directed toward the right ventricle (RV) and its interaction with the pulmonary circulation [6]. Right v ent r i cul a r dy s func t ion and pulmona r y hypertension frequently coexist in HF and are powerful determinants of symptoms, exercise intolerance, and adverse outcomes [7-9]. However, isolated assessment of RV systolic function or pulmonary artery pressure provides only a partial representation of right heart performance, as RV contractility is intrinsically load dependent [10]. Consequently, parameters that fail to integrate RV function with its afterload may underestimate disease severity and prognostic risk [11]. The concept of right ventricular–pulmonary arterial (RV–PA) coupling has therefore emerged as a physiologically grounded framework to evaluate the ability of the RV to adapt to increased pulmonary vascular load [12,13]. Noninvasively, RV–PA coupling can be approximated using the ratio between TAPSE and sPAP, a simple echocardiographic index that integrates RV longitudinal systolic performance with pulmonary afterload [14]. TAPSE/sPAP has been proposed as a surrogate of RV–PA interaction and has demonstrated prognostic relevance in selected populations, including pulmonary hypertension [15,16] and specific HF subgroups [17,18].

METHODS

Search strategy A comprehensive literature search was independently performed by two investigators (A.S. and G.F.G.) using the PubMed, Scopus, and EMBASE databases from inception through January 2025. The search strategy was designed to identify studies evaluating the prognostic role of right ventricular–pulmonary arterial coupling assessed by echocardiography in patients with heart failure. The following terms and their combinations were used: “heart failure”, “acute heart failure”, “chronic heart failure”, “TAPSE”, “tricuspid annular plane systolic excursion”, “systolic pulmonary artery pressure”, “sPAP”, “TAPSE/sPAP”, “RV–PA coupling”, “right ventricular–pulmonary arterial coupling”, “prognosis”, “mortality”, and “outcomes”. The search was restricted to full-text articles published in English. Reference lists of eligible studies and relevant reviews were manually screened to identify additional pertinent publications.

Participant or population The included populations encompassed a wide spectrum of heart failure phenotypes and clinical settings. These ranged from acute and chronic heart failure cohorts with preserved, mildly reduced, or reduced ejection fraction to more specific populations, such as cardiac amyloidosis, heart failure secondary to severe aortic stenosis, degenerative or secondary mitral regurgitation, advanced heart failure referred for transplantation, and post–heart transplant recipients. In addition, one large contemporary study focused specifically on older patients hospitalized for acute heart failure with preserved ejection fraction, further expanding the representation of acute care settings and elderly populations. Both ischemic and non-ischemic etiologies were represented, although several studies did not stratify outcomes according to heart failure etiology.

Intervention The aim of the present systematic review was to comprehensively evaluate the prognostic value of the echocardiographic TAPSE/ sPAP ratio in patients with heart failure. By synthesizing data from available observational studies, we sought to (i) characterize the clinical and echocardiographic profiles of patients in whom TAPSE/sPAP has been assessed, (ii) examine the consistency of its association with adverse outcomes across diverse HF phenotypes and settings, and (iii) identify convergent prognostic thresholds that may support its use as a practical and reproducible marker of RV–PA uncoupling in heart failure.

Comparator N/A.

Study designs to be included Observational Cohort and Cross-Sectional Studies.

Eligibility criteria Studies were eligible for inclusion if they enrolled adult patients with heart failure, regardless of phenotype or clinical setting, assessed right ventricular–pulmonary arterial coupling using the echocardiographic TAPSE/sPAP ratio, and evaluated its association with prognostic out come such as all-cause mortality, cardiovascular mortality, heart failure hospitalization, or composite endpoints. Both prospective and retrospective observational cohort studies were considered eligible. Studies were excluded if they were non-clinical, experimental, or animal studies; case reports, small case series, conference abstracts, editorials, letters without original data, narrative reviews, or meta-analyses; studies assessing RV–PA coupling exclusively by cardiac magnetic resonance imaging (CMR) or other non-echocardiographic modalities; if they did not report clinical outcomes; if echocardiographic data were insufficient to derive TAPSE/sPAP; or if they represented duplicate reports from the same study population, in which case the most complete or recent publication was retained.

Information sources A comprehensive literature search was independently performed by two investigators (A.S. and G.F.G.) using the PubMed, Scopus, and EMBASE databases from inception through January 2026.

Main outcome(s) To evaluate the prognostic role of TAPSE/sPAP ratio in various cohorts of HF patients.

Additional outcome(s) To (i) characterize the clinical and echocardiographic profiles of patients in whom TAPSE/sPAP has been assessed, (ii) examine the consistency of its association with adverse outcomes across diverse HF phenotypes and settings, and (iii) identify convergent prognostic thresholds that may support its use as a practical and reproducible marker of RV–PA uncoupling in heart failure.

Data management Two investigators (A.S. and G.F.G.) independently screened all retrieved records by title and abstract, followed by full-text assessment of potentially eligible studies according to the predefined inclusion and exclusion criteria. Disagreements regarding study eligibility or data extraction were resolved through discussion and consensus, and when agreement could not be reached, a third reviewer was consulted for adjudication. Data extraction was independently performed by the same investigators using a predefined standardized form. Extracted data included study characteristics (author, year, country, design, sample size, and follow-up duration), patient demographics and heart failure phenotype, clinical variables and comorbidities, laboratory data, pharmacological treatment, echocardiographic parameters with particular focus on TAPSE, sPAP, and the TAPSE/ sPAP ratio, as well as reported prognostic outcomes, effect estimates, and proposed TAPSE/ sPAP cut-off values. A third investigator (G.L.N.) reviewed the extracted data to ensure accuracy and resolve any discrepancies.

Quality assessment / Risk of bias analysis The risk of bias of the included studies was independently assessed by two reviewers (A.S. and G.L.N.) using the National Institutes of Health (NIH) Quality Assessment Tool for Observational Cohort and Cross-Sectional Studies. Each study was evaluated across the 14 methodological domains of the tool and classified as having good, fair, or poor methodological quality. Disagreements were resolved by consensus.

Strategy of data synthesis Continuous and categorical variables extracted from the included studies were summarized using pooled descriptive statistics. Given the expected non-normal distribution of most clinical and echocardiographic parameters, central tendency was expressed as weighted medians with corresponding interquartile ranges. Study-level estimates were weighted by sample size to account for differences in cohort size and to limit the influence of small exploratory studies. When variables were reported exclusively as mean ± standard deviation, the reported mean value was retained for descriptive purposes, as transformation to medians was not feasible without access to individual-level data. Categorical variables were summarized as weighted proportions and expressed as weighted percentages. Missing data were handled on a per-variable basis, with pooled estimates calculated only from studies reporting the relevant parameter and without imputation of missing values. For each variable, both the total sample size and the number of contributing studies were reported to ensure transparency regarding data availability and to facilitate interpretation of variability across studies. Between-study heterogeneity was explored descriptively by systematically examining differences in patient characteristics, heart failure phenotype, clinical setting, study design, timing of TAPSE/sPAP assessment, and outcome definitions. Formal statistical measures of heterogeneity were not calculated, as no pooled effect estimates were derived. In accordance with current recommendations for systematic reviews of observational studies, no formal meta-analysis was performed.

Subgroup analysis N/A.

Sensitivity analysis NR.

Language restriction No.

Country(ies) involved Italy.

Keywords heart failure; right ventricular–pulmonary arterial coupling; TAPSE/sPAP; echocardiography; right ventricular function; prognosis.

Contributions of each author

Author 1 - Andrea Sonaglioni - Author 1 drafted the manuscript. Email: sonaglioniandrea@gmail.com

Author 2 - Giulio Francesco Gramaglia – The author contributed to the Search Strategy. Email: giulio.gramaglia@unimi.it

Author 3 - Massimo Baravelli - Supervision. Email: massimo.baravelli@multimedica.it

Author 4 - Michele Lombardo - Supervision. Email: michele.lombardo@multimedica.it

Author 5 - Gian Luigi Nicolosi - The authors revised the original manuscript. Email: gianluigi.nicolosi@gmail.com

Author 6 - Sergio Harari - Supervision. Email: sergio.harari@unimi.it

References

1. Savarese, G.; Becher, P.M.; Lund, L.H.; Seferovic, P.; Rosano, G.M.C.; Coats, A.J.S. Global burden of heart failure: A comprehensive and updated review of epidemiology. *Cardiovasc. Res.* **2023**, 118, 3272–3287. doi:10.1093/cvr/cvac013.
2. Nguyen, A.H.; Hurwitz, M.; Abraham, J.; Blumer, V.; Flanagan, M.C.; Garan, A.R.; Kanwar, M.; Kataria, R.; Kennedy, J.L.W.; Kochar, A.; et al. Medical management and device-based therapies in chronic heart failure. *J. Soc. Cardiovasc. Angiogr. Interv.* **2023**, 2, 101206. doi:10.1016/j.jscai.2023.101206.
3. Sengupta, P.P.; Kramer, C.M.; Narula, J.; Dilsizian, V. The potential of clinical phenotyping of heart failure with imaging biomarkers for guiding therapies: A focused update. *JACC Cardiovasc. Imaging* **2017**, 10, 1056–1071. doi:10.1016/j.jcmg.2017.07.001.
4. Ibrahim, N.E.; Januzzi, J.L. Established and emerging roles of biomarkers in heart failure. *Circ. Res.* **2018**, 123, 614–629. doi:10.1161/CIRCRESAHA.118.312706.
5. Bayes-Genis, A.; Aimo, A.; Jhund, P.; Richards, M.; de Boer, R.A.; Arfsten, H.; Fabiani, I.; Lupón, J.; Anker, S.D.; González, A.; et al. Biomarkers in heart failure clinical trials: A review from the Biomarkers Working Group of the Heart Failure Association of the European Society of Cardiology. *Eur. J. Heart Fail.* **2022**, 24, 1767–1777. doi:10.1002/ejhf.2675.
6. Pinsky, M.R. The right ventricle: Interaction with the pulmonary circulation. *Crit. Care* **2016**, 20, 266. doi:10.1186/s13054-016-1440-0.
7. Ghio, S.; Gavazzi, A.; Campana, C.; Inserra, C.; Klersy, C.; Sebastiani, R.; Arbustini, E.; Recusani, F.; Tavazzi, L. Independent and additive prognostic value of right ventricular systolic function and pulmonary artery pressure in patients with chronic heart failure. *J. Am. Coll. Cardiol.* **2001**, 37, 183–188. doi:10.1016/S0735-1097(00)01102-5.
8. Mohammed, S.F.; Hussain, I.; AbouEzzeddine, O.F.; Takahama, H.; Kwon, S.H.; Forfia, P.; Roger, V.L.; Redfield, M.M. Right ventricular function in heart failure with preserved ejection fraction: A community-based study. *Circulation* **2014**, 130, 2310–2320. doi:10.1161/CIRCULATIONAHA.113.008461.
9. Bernardo, R.J.; Haddad, F.; Couture, E.J.; Hansmann, G.; de Jesus Perez, V.A.; Denault, A.Y.; de Man, F.S.; Amsallem, M. Mechanics of right ventricular dysfunction in pulmonary arterial hypertension and heart failure with preserved ejection fraction. *Cardiovasc. Diagn. Ther.* **2020**, 10, 1580–1603. doi:10.21037/cdt-20-479.

10. Haddad, F.; Doyle, R.; Murphy, D.J.; Hunt, S.A. Right ventricular function in cardiovascular disease, part II: Pathophysiology, clinical importance, and management of right ventricular failure. *Circulation* **2008**, 117, 1717–1731. doi:10.1161/CIRCULATIONAHA.107.653584.
11. Guazzi, M.; Naeije, R. Right heart phenotype in heart failure with preserved ejection fraction. *Circ. Heart Fail.* **2021**, 14, e007840. doi:10.1161/CIRCHEARTFAILURE.120.007840.
12. Tedford, R.J.; Hassoun, P.M.; Mathai, S.C.; Girgis, R.E.; Russell, S.D.; Thiemann, D.R.; Cingolani, O.H.; Mudd, J.O.; Borlaug, B.A.; Redfield, M.M.; et al. Pulmonary capillary wedge pressure augments right ventricular pulsatile loading. *Circulation* **2012**, 125, 289–297. doi:10.1161/CIRCULATIONAHA.111.051540.
13. Sanz, J.; Sánchez-Quintana, D.; Bossone, E.; Bogaard, H.J.; Naeije, R. Anatomy, function, and dysfunction of the right ventricle: JACC state-of-the-art review. *J. Am. Coll. Cardiol.* **2019**, 73, 1463–1482. doi:10.1016/j.jacc.2018.12.076.
14. Tello, K.; Wan, J.; Dalmer, A.; Vanderpool, R.; Ghofrani, H.A.; Naeije, R.; Roller, F.; Mohajerani, E.; Seeger, W.; Herberg, U.; et al. Validation of the tricuspid annular plane systolic excursion/systolic pulmonary artery pressure ratio for the assessment of right ventricular–arterial coupling in severe pulmonary hypertension. *Circ. Cardiovasc. Imaging* **2019**, 12, e009047. doi:10.1161/CIRCIMAGING.119.009047.
15. Vanderpool, R.R.; Pinsky, M.R.; Naeije, R.; Deible, C.; Kosaraju, V.; Bunner, C.; Mathier, M.A.; Lacomis, J.; Champion, H.C.; Simon, M.A. RV–pulmonary arterial coupling predicts outcome in patients referred for pulmonary hypertension. *Heart* **2015**, 101, 37–43. doi:10.1136/heartjnl-2014-306142.
16. Tello, K.; Axmann, J.; Ghofrani, H.A.; Naeije, R.; Narcin, N.; Rieth, A.; Seeger, W.; Gall, H.; Richter, M.J. Relevance of the TAPSE/PASP ratio in pulmonary arterial hypertension. *Int. J. Cardiol.* **2018**, 266, 229–235. doi:10.1016/j.ijcard.2018.01.053.
17. Guazzi, M.; Naeije, R.; Arena, R.; Corrà, U.; Ghio, S.; Forfia, P.; Rossi, A.; Cahalin, L.P.; Bandera, F.; Temporelli, P. Echocardiography of right ventriculoarterial coupling combined with cardiopulmonary exercise testing to predict outcome in heart failure. *Chest* **2015**, 148, 226–234. doi:10.1378/chest.14-2065.
18. Bandera, F.; Barletta, M.; Fontana, M.; Boveri, S.; Ghizzardi, G.; Alfonzetti, E.; Ambrogi, F.; Guazzi, M. Exercise-induced mitral regurgitation and right ventricle–pulmonary circulation uncoupling across heart failure phenotypes. *Am. J. Physiol. Heart Circ. Physiol.* **2021**, 320, H642–H653. doi:10.1152/ajpheart.00507.2020.
